# Indoor air pollution and cognitive function among older adults in India: a multiple mediation approach through depression and sleep disorders

**DOI:** 10.1186/s12877-024-04662-6

**Published:** 2024-01-22

**Authors:** Subhojit Shaw, Sampurna Kundu, Aparajita Chattopadhyay, Smitha Rao

**Affiliations:** 1 Department of Population and Development, International Institute for Population Sciences, Deonar, Mumbai, 88 India; 2 Centre of Social Medicine & Community Health, Jawaharlal Nehru University, New Delhi, Delhi, 110067 India; 3 College of Social Work, The Ohio State University, Columbus, 43214 USA

**Keywords:** Indoor air pollution, Cognition, Depression, Sleep disorders, Multiple mediation

## Abstract

**Background:**

Studies across multiple countries reveal that depression and sleep disorders can lead to cognitive decline. This study aims to speculate on the effect of different sources of indoor air pollution on cognition and to explore the mediation effect of depression and sleep disorders on cognition when exposed to indoor air pollution. We hypothesize that an older adult experiences higher cognitive decline from indoor pollution when mediated by depression and sleep disorders.

**Methods:**

We use data from Longitudinal Aging Study in India (LASI), 2017–2018, and employ a multiple mediation model to understand the relationship between indoor air pollution and cognition through sleep disorders and depression while adjusting for possible confounders. Sensitivity analysis was applied to see the effect of different sources of indoor pollution (cooking fuel, indoor smoke products, and secondhand smoke) on cognitive performance.

**Results:**

The effect of three sources of indoor pollutants on cognition increased when combined, indicating stronger cognitive decline. Unclean cooking practices, indoor smoke (from incense sticks and mosquito coils), and secondhand smoke were strongly associated with sleep disorders and depression among older adults. Indoor air pollution was negatively associated with cognitive health (β= -0.38) while positively associated with depression (β= 0.18) and sleep disorders (β= 0.038) acting as mediators. Sensitivity analysis explained 45% variability while adjusting for confounders.

**Conclusion:**

The study lays a foundation for future investigations into the nexus of indoor pollution and mental health. It is essential to formulate policies to reduce exposure to varying sources of indoor air pollutants and improve screening for mental health services as a public health priority.

**Supplementary Information:**

The online version contains supplementary material available at 10.1186/s12877-024-04662-6.

## Introduction

Air pollution is a serious environmental health concern worldwide. Combustion of fuelwood, coal, agricultural residue, cow dung cake, incense sticks, mosquito coils, and second-hand smoke are some of the most important contributors to indoor air pollution [1, 2]. Empirical studies suggest harmful air pollutants may reach beyond the cerebral blood barrier and cause psychiatric disorders, including neurodegenerative diseases, sleeplessness, depression, and poor cognition [3, 4]. Older adults are more vulnerable and exposed to short-term air pollution, often because of weakened immune functions. Long-term air pollution exposure has been linked to various neurological disorders, including Parkinson's disease and Alzheimer's disease, occurring more often among older adults [5].

Particulate matter, nitrogen dioxide, and sulfur dioxide are the primary components of air pollution in countries like India which suffers hugely from indoor as well as outdoor pollution [6]. In India, indoor air pollution caused 0.61 million deaths and 4.5 percent of disability-adjusted life years (DALY) in 2019 [7]. Residential solid-fuel combustion primarily consists of black and organic carbon, with traces of various hazardous compounds and soot. Although sources and their magnitudes differ by geography, household solid fuel use is the largest across Asia, especially in South Asia [8]. Indoor air pollution is responsible for ischemic heart diseases (27%), pneumonia (27%), chronic obstructive pulmonary diseases (20%), stroke (18%), and lung cancer (8%), accounting for approximately 4 million deaths worldwide [9]. In addition to physical health impairment, studies have suggested that indoor air pollution contributes to cognitive decline and may also result in mental disorders [10, 11].

### Sources of indoor air pollution and impact on cognitive function in India

The principal sources of indoor air pollution in India are combustion, building material, and bioaerosols. Studies have found that the residential sector contributes 20–50% of indoor pollution [12]. In India, unsafe cooking practices comprise firewood (45%); cow dung cake, coal, charcoal (10.4%) and kerosene (2.9%) [13]. Urban populations are predominantly exposed to vehicular and industrial exhausts, which directly and indirectly contributes to public health risks to all households [12, 14]. The established risk of respiratory disease, cardiovascular diseases, birth defects, blindness, depression and cognitive dysfunction among adult and older adults is a concern in low- and middle-income countries (LMIC) where biomass fuels are used for cooking and heating [1, 15]. Both social and environmental factors influence cognition, and literature suggests that indoor air pollution is widespread in low-and-middle-income countries [15]. In a poorly ventilated house, polluted air is inhaled through our circulatory system, eventually damaging the lungs, heart, and brain, causing inflammation and neuronal dysfunction [16]. The possible biological pathway is that air particles could get directly to the brain through the olfactory bulb and affect the central nervous system with oxidative stress and neuroinflammation [17]. Evidence suggests that olfactory bulbs loaded with particulate matter and impaired olfactory function are precursors to dementia and Alzheimer's disease [18]. Experimental studies in China suggest that indoor air pollution has an adverse effect on older adult cognition, especially for short-term memory loss and mathematical reasoning abilities [10, 11]. A similar study from Mexico reported that persons aged 50 and above exposed to indoor air pollution had lower cognitive outcomes [1, 19]. This evidence base necessitates an empirical study investigating the influence of indoor air pollution from solid fuel on cognitive decline to derive insights to center the critical issue of the mental health of older adults in India.

### Mediating role of depression between air pollution and cognition

Depression is a pressing public health challenge affecting people of all ages and a leading cause of morbidity. Pollutants like particulate matter (PM_2.5_) cause cell cycle arrest and death in neurons, with oxidative stress and DNA damage, resulting in brain degeneration [17, 20]. Depressive symptoms correlate negatively with poor cognitive function [21]. Cognitive impairments interfere with one's ability to think or focus, including indecisiveness, and memory loss, all of which are criterion items for diagnosing depression [22]. Patients with depression report trouble distinguishing between similar or identical objects and short-term memory loss [23, 24]. Reinforcing neurological evidence of air pollution exposure and cognitive outcomes of anxiety and depression, we posit that depression could mediate associations between air pollution and cognitive impairment.

### Mediating role of sleep disorders between air pollution and cognition

The International Classification of Sleep Disorders (ICSD-3) identifies sleep-related breathing disorders, central disorders of hypersomnolence, circadian rhythm sleep-wake disorders, and sleep-related movement, parasomnias, insomnia, and other sleep disorders as the major categories of sleep-related disorders. Increases in PM_10_ are associated with decreasing sleep efficiency [25]. There is also mechanistic evidence between air pollution and sleep disorders through alterations in inflammatory cytokines in the blood [26]. Yu et al., (2020) found a significant association between solid fuel use and shorter and restless sleep days among Chinese older adults. Difficulties with sleep are common among older adults and are caused by multimorbidity, the aging process, and increased medication, among other factors [27]. Changes in sleep structure with aging are linked to cognitive decline. Aging is linked to decreased performance across various cognitive activities, including information processing, perceptual speed, executive functioning, concentration and attention, inhibitory functioning, and memory [28]. Treating sleep disorders may enhance cognitive performance and overall quality of life. Understanding the possibility of an individual's health, social, and environmental factors influencing sleep disturbances, we hypothesize that sleep disorders would mediate the association between air pollution and cognitive impairment.

### Multiple mediation between air pollution and cognition through depression and sleep problems

Some of the most prevalent mental and neurological diseases among older adults are cognitive decline, sleeplessness, and depression [29]. Scholars have linked long-term air pollution exposure to depression, sleep problems, and cognitive loss [30, 31]. In rural India, fuel choices are limited primarily to biomass sources such as fuelwood, agricultural residue, kerosene, and cow dung cake for cooking and heating [32]; only 19% of rural families use liquefied petroleum gas (LPG), a cleaner choice, as their primary cooking fuel, while most others cook using biomass fuels [33]. Per the National Sample Survey (NSS 68^th^ Round), 56% of rural households relied on solid fuels during 2011-2012. Thereafter, National Family Health Surveys (NFHS-5), 2019-2021, reported that 40.6% of households use solid fuels for cooking and 24.5% smoke inside the home daily. Despite government programs providing LPG to people below the poverty line under *Pradhan Mantri Ujjwala Yojana* (PMUY) 2016, India's rural areas still primarily rely on unhealthy cooking practices.

**Fig. 1:**
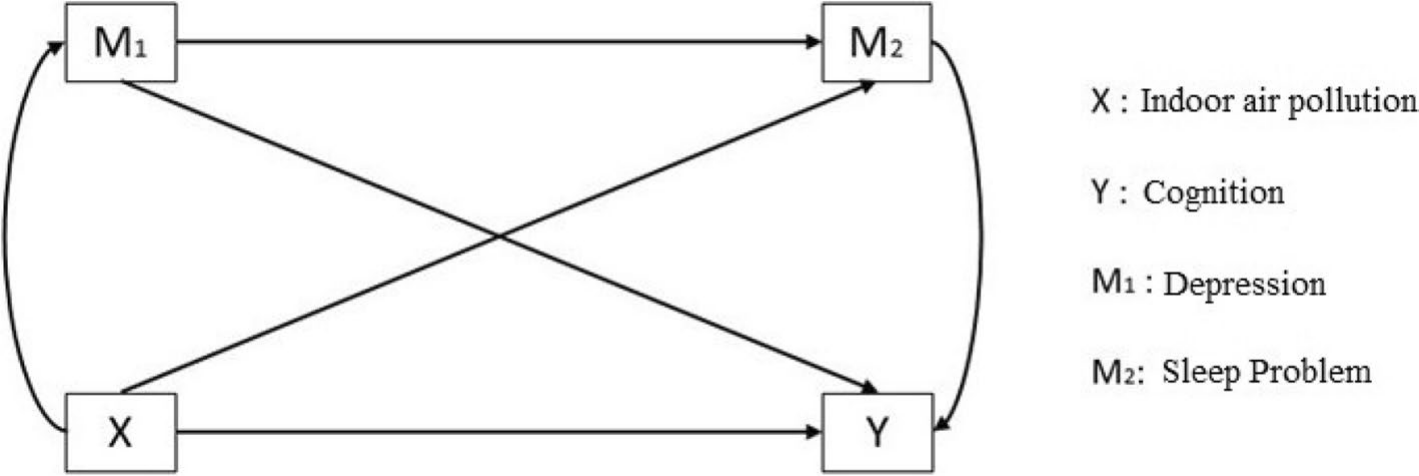
Proposed path diagram of the multiple-mediation model for the study of X: independent variable (indoor air pollution); Y: outcome variable (cognition); M_1_: mediator (depression); M_2_: mediator (sleep problem)

Few studies in India have examined how indoor air pollution affects cognitive function through other underlying psychological/neurological mechanisms [1, 15]. We include multiple sources of air pollution and explore this gap in literature by reconnoitering sleep disorders and depression as mediators between air pollution and cognition using nationally representative data. Mediation models are well-established and used in various fields, including psychology, gerontology, behavioral science, genetic epidemiology, preventative research, and political communication research [4, 34]. We speculate that depression and sleep disorders may mediate the association between air pollution exposure and cognition. Compared to single mediation models, the multiple mediation model (Fig. 1) simulates several mechanisms to examine how indoor air pollution relates to cognitive health. We propose to decompose indoor air pollution's direct and indirect effects on cognitive health through sleep problems and depression while adjusting for possible confounders.

A comprehensive assessment of indoor air pollution and associated health impacts is necessary to strengthen public health policy in India. We focus on the association between air pollution and cognitive decline, offering a multiple mediation approach to investigate this relationship.

## Material and methods

Our study utilizes data from Longitudinal Aging Study in India (LASI), Wave1, collected in 2017-18. LASI is in harmony with the Global Health and Retirement Study (HRS). LASI collected information on physical and psychological health, social security, and family dynamics of adults aged 45 and above, including their spouses residing in the same household (irrespective of age). The survey used a multi-stage stratified probability cluster sampling method, in which a three-stage and four-stage sampling design was used for rural and urban areas, respectively. The indicators of indoor air pollution from the household dataset were merged with the individual dataset to get all relevant variables yielding a sample size of 72,250 individuals.

### Key Measures

#### Dependent variable

We measured cognitive health under five domains: memory, orientation, arithmetic, executive functioning, and object naming, and constructed a composite index as a part of the regular cognition module of the Health and Retirement Study (HRS). Overall, cognitive scores ranged from 3-43, with higher scores indicating better cognitive function [35].

#### Independent variable

Indoor air pollution was measured by poor cooking practices (use of unclean fuel inside home and cooking in traditional chullah/stove or open fire without ventilation.), use of smoke products indoors (use of incense sticks, mosquito coil, and fast card: paper-based mosquito repellents are paper cards infused with mosquito repellent chemicals that activate on burning), and second-hand smoking (smoking inside home by any household member). For index construction, we used factor analysis using principal component extraction to explore the underlying structure of the variables measuring the pollution dimension. The factor model fit was examined using the Kaiser Meyer Olkin (KMO) test, which measures sampling adequacy, and Bartlett’s test of sphericity. We built three indices to investigate the effect of the changes in index constructs:

- Indoor Pollution (IP1) – Poor cooking practices
- Indoor Pollution (IP2) - Poor cooking practices + indoor smoke product usage
- Indoor Pollution (IP3) - Poor cooking practices + indoor smoke product usage + second-hand smoke

#### Mediators

The two mediators in our study were sleep problems and depression. To measure depressive symptoms, ten items from the Centre for Epidemiologic Studies Depression (CES-D) scale were considered [36]. Items were divided into seven negative and three positive symptoms, with a score ranging from 0-10. Negative symptoms were scored in ascending order, whereas scoring was reversed for positive symptoms; therefore, higher scores indicate severe depressive symptoms [37]. Sleep disorders or problems are measured in LASI using four indicators, including trouble falling asleep, waking up in the middle of the night, waking up too early, and feeling unrest during daytime. The categories occasionally (3-4 nights/ week) and frequently (5 or more nights per week) were considered sleep problems.

#### Confounders

We adjusted for eight socio-demographic variables. Sex of the respondent was dichotomized as 'male' and 'female'. Age was a continuous variable consisting of adults aged 45 or more (including spouses irrespective of age) [37]. Residence was coded as 'rural' and ‘urban.' Education was a continuous variable indicated by years of schooling ranging from 0-26 years. Religion was coded as 'Hindu' and 'Others,' and the various social caste groups were coded as 'Scheduled Caste' (SC)/Scheduled Tribe' (ST) and ‘Others’, where SC/ST population is largely deprived of privileges, historically and socially marginalized, and vulnerable. Marital status was coded as 'Married' and ‘Not married.’ A structured set of 11 and 29 questions on food and non-food expenses were asked to measure the economic condition. Monthly Per capita Consumption Expenditure (MPCE) was calculated and used as the summary measure of consumption. Food expenditure was collected over a seven-day reference period, whereas non-food expenditure was collected over 30- and 365-day reference period. The 30-day reference period was used to standardize food and non-food expenditures. The variable was then coded into five quintiles: poorest, poor, middle, rich, and richest.

## Statistical methods

### Descriptive and bivariate analyses

Key variables were first described using summary statistics: mean, standard deviations (continuous variables), frequency distribution, and percentages (categorical variables). The Table S1 in supplementary material provides the detailed descriptive statistics of all the variables. Bivariate analyses examined the association between independent variable (*X*), mediators (*M1 and M2*), possible confounders, and dependent variable cognition (*Y*). Independent t-tests were employed for categorical variables with two categories, one-way ANOVA F-test for more than two categories, and correlation tests were used for continuous variables.

### Mediation analysis

The multiple mediation model, suggested by Preacher and Hayes [38, 39], accommodates both mediation factors simultaneously while adjusting for possible confounders. Mediation processes are framed with total effects of the independent variable (X) on the dependent variable (Y), with a transition of mediating factor (M). The effect of X on Y is the direct effect (DE), and when the effect of X is transmitted through M on Y, it is the indirect effect (IE). Therefore, the total effect (TE) is decomposed into direct and indirect effects [40]. To test the mediation hypothesis, we followed guidelines from Preacher & Hayes (2008). We built three models analyzing indoor air pollution using independent variables as IP1, IP2, and IP3 as Model 1, Model 2, and Model 3, respectively [41].

The model analyzes the relationship between IP1/IP2/IP3 (*X*) and cognition (*Y*) mediated through depression (*M1*) and sleep problems (*M2*) with the following steps: (i) regressing *Y* on *X*; (ii) regressing *M1* on *X*; and (iii) regressing M2 on X and M1; and (iv) regressing Y on *X*, *M1,* and *M2*. There are three requirements for mediation to exist - *a*_*1*_*,a*_*2*_ are significant, indicating *X* is related to *M1* (Equations 2, 3); *b*_*1*_*, b*_*2*_ is significant, indicating *M1* and *M2* are associated with *Y* (Equation 3); and if indirect effect *c'* is not significant; if significant, then it indicates partial mediation (Fig. 1). This model provides direct and indirect paths among variables while controlling for confounders.

*Estimated effect of X on Y through M1 and M2*

1 $$Y={i}_{y}+cX+{\epsilon }_{1}$$

2 $$M1={i}_{m1}+{a}_{1}X+{\epsilon }_{2}$$

3 $$M2={i}_{m2}+{a}_{2}X+{a}_{3}M1+{\epsilon }_{3}$$

4 $$Y={i}_{y{\prime}}+{c}{\prime}X+{b}_{1}M1+{b}_{2}M2+{\epsilon }_{4}$$

where, iy, im1, im2, iy' are intercepts; a1 is the effect of X on M1; a2 is the effect of X on M1; a3 is the effect of M1 on M2; b1 and b2 are the effects of M1 and M2 on Y; c and c' are the direct and indirect effects of X on Y.

Mediation analysis was performed in SPSS based on 5000 bootstrapped samples (bias-corrected) and sorting them to yield 95% confidence intervals. First, we regressed Y over IP1, followed by IP2 and IP3, which we used to compare and observe whether changes in indoor pollution construction yielded different results and choose the most informative aspects. We also included a spatial component to visualize the distribution of indoor air pollution, cognitive function, depression, and sleep problems across India.

## Results

Table S1 (appendix) shows key summary statistics for variables in our analysis. Our sample comprised 42% males and 58% females, with a mean age of 58.57. Most respondents belonged to rural areas (68.2%), Hindu religion (81.92%), and other castes (72.32%). Around 75.6% of respondents were married. The average years of schooling in the sample was 4.06. Indoor air pollution measured by IP1 (poor cooking practices), IP2 (poor cooking practices + indoor smoke product usage), and IP3 (poor cooking practices + indoor smoke product usage + second-hand smoke) had a mean score of 3.65, 2.12, and 2.06, respectively. The dependent variable, cognitive health score, ranged between 3 to 43, with a mean score of 25.38. The depression score had a mean of 2.94 and ranged from 0 to 10. The second mediator, sleep problems, had a mean score of 0.79 and ranged between 0-4.

### Cognitive score by selected characteristics

The mean cognitive score was higher among males (27.66), respondents in urban areas (28.38), with the highest level of education (32.35), respondents who were unmarried (25.78), not belonging to lower caste (26.68), and in highest MPCE quintile (27.74) (Table 1). There was a decline in cognitive function with increasing age (*r*=-0.26). Years of schooling had a significant and positive correlation with cognitive function (*r*=0.63). Depression (*r*=-0.16) and sleep problems (*r*=-0.12) were significantly correlated with cognition. The indoor air pollution indices indicated by poor cooking practices (IP1) (*r*-0.23); poor cooking practices with indoor smoke product use (IP2) (*r*=-0.18); and poor cooking practices with indoor smoke product use and secondhand smoke (IP3) (*r*=-0.17), all were significantly negatively correlated with cognition (Table 1).

**Table 1:** Bivariate analyses of covariates and mediators with cognition as dependent variable

**Covariates**		**Cognition (Y)**		
		*Mean (S.D)*	*Test*	*p-value*
Gender	Male	27.66(0.04)	t=60.28	<0.001
	Female	24.53(0.04)		
Age			r=-0.26	<0.001
Residence	Rural	24.43(0.03)	t=-74.67	<0.001
	Urban	28.38(0.04)		
Education	Years of schooling		r=0.63	<0.001
Religion	Hindu	25.97(0.03)	t=-6.12	<0.001
	Others	25.60(0.05)		
Marital status	Currently married	26.63(0.03)	t=-53.29	<0.001
	Not married	23.12(0.06)		
Caste	SC/ST	24.24(0.05)	t=43.52	<0.001
	Others	26.68(0.03)		
MPCE	Poorest	24.06(6.7)	F=567.90	<0.001
	Poorer	25.04(6.66)		
	Middle	25.78(6.77)		
	Richer	26.58(6.7)		
	Richest	27.74(6.72)		
Indoor air pollution	IP1		r=-0.23	<0.001
	IP2		r= -0.18	<0.001
	IP3		r=-0.17	<0.001
Depression (M1)			r=-0.16	<0.001
Sleep problems (M2)			r=-0.12	<0.001

### Spatial distribution of indoor air pollution and mental health indicators

**Fig 2:**
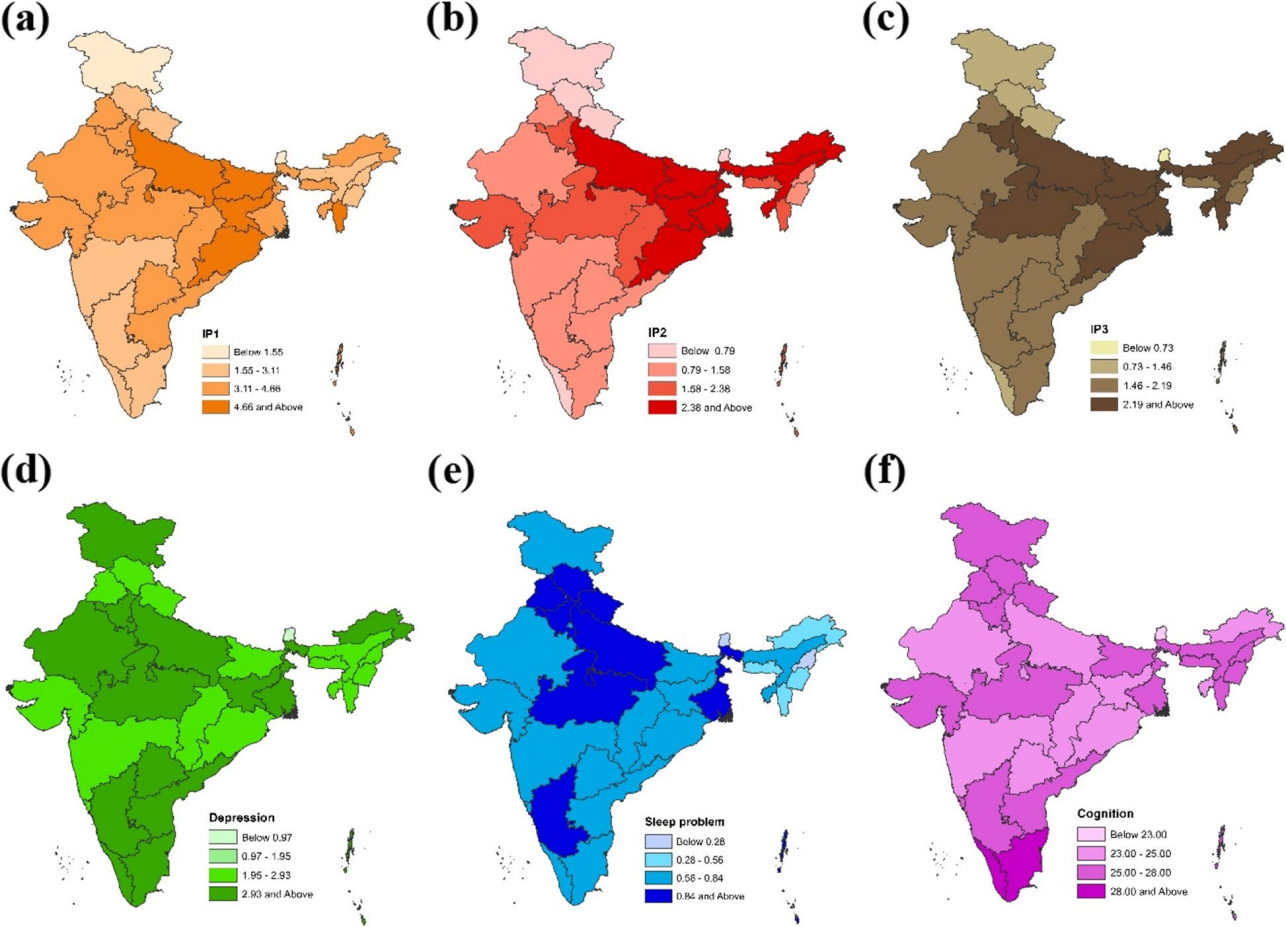
Spatial distribution of mean score: (**a**) IP1- poor cooking practices (**b**) IP2- poor cooking practices + indoor smoke product usage (**c**) IP3- poor cooking practices + indoor smoke product usage + second-hand smoke (d) Sleep problems (**e**) Depression (**f**) Cognition across States/UTs of India, LASI (2017-2018). *Note:* Sikkim not included

Figure 2 a, b, and c presents the spatial distribution of various indoor air pollution sources across India. Higher intensity of the color shows higher indoor pollution levels. Northern and northeastern states showed higher levels of indoor air pollution from sources like unsafe cooking practices, indoor smoke product usage, and secondhand smoke exposure. Depression was higher in southern, northern, and parts of eastern states in India (Fig. 2d); higher intensity of the color shows higher depression levels. Sleep problems were higher in the northern states (Fig. 2e); the higher intensity of the color shows higher sleep problems among older adults, although Karnataka (south) and West Bengal (east) emerged as outliers with higher sleep-related problems. A high cognition score, indicating better mental health condition, was observed in the southern states; higher intensity of the color shows a better mean cognitive level, i.e., Kerala and Tamil Nadu (Fig. 2f).

**Fig 3:**
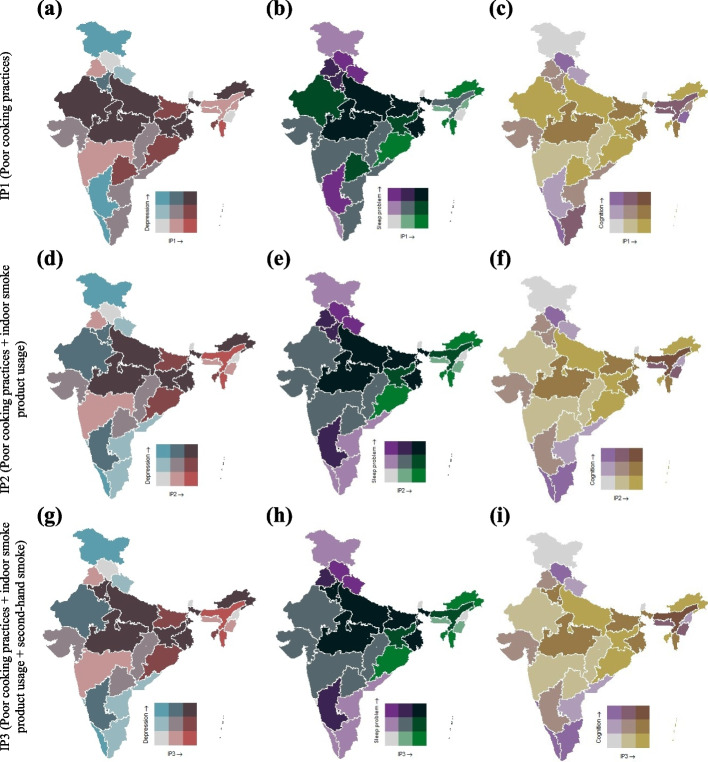
Bivariate plot for sleep problems, depression, and cognition with **a**) IP1- poor cooking practices (**b**) IP2- poor cooking practices + indoor smoke product usage (**c**) IP3- poor cooking practices + indoor smoke product usage + second-hand smoke across different State/UTs of India, LASI (2017-2018). *Note:* Sikkim not included

Figure 3 depicts the spatial bivariate relationship between indoor air pollution with three mental health conditions, i.e., depression, sleep problems, and cognition. Excepting maps indicating the relationship between cognition and pollution, in all other maps (Fig. 3a, b, d, e, g, h), dark brown or dark green reflects high pollution and mental health issues. While for maps on cognition and pollution (Fig. 3 c, f, i), the yellow color reflects high pollution with low cognition level. High depression levels are associated with increased exposure to indoor air pollution from unsafe cooking practices in northern states like Rajasthan, Uttar Pradesh, Madhya Pradesh, and a few states in India's east and northeast (Fig. 3 a, d, g). For instance, higher levels of indoor air pollution are associated with higher levels of sleep problems in the northern states of Uttar Pradesh, Madhya Pradesh, Bihar, and West Bengal of East India (Fig. 3b, e, h). In Fig. 3 c, higher levels of indoor air pollution are associated with lower cognitive scores in northern states such as Uttar Pradesh and Rajasthan and eastern states of Jharkhand, Odisha, and some northeastern states. Southern states, except Telangana, showed higher cognitive scores with lower indoor pollution. Overall, southern states fared better than the rest of India regarding lower indoor pollution and cognitive decline.

### Mediation effects

Table 2 provides estimated coefficients with statistical significance and standard errors from the mediation analysis. In the first model, indoor air pollution measured in terms of poor cooking practices (IP1) was negatively associated with cognition (β:-0.394; p<0.001). In Model 1, where depression and sleep problems were considered as mediators, depression was negatively associated with cognitive health (β:-0.237; p<0.001), and sleep problems were also negatively associated (β:-0.158; p<0.001) with cognitive health. On the other hand, the effect of IP1 on cognition was statistically significant and positively associated with depression (β:0.158; p<0.001) and sleep problems (β:0.011; p<0.01). The indirect effect of IP1 on cognition, mediated by depression and sleep problems, was -0.043 at (p<0.01), which indicated partial mediation effects of depression and sleep problems between indoor air pollution and cognitive health. Model 1 explained 45.6% of the variability in cognitive health. Similarly, in model 2, indoor air pollution indicated by poor cooking practices along with indoor smoke product use (IP2) showed a negative association with cognition (β:-0.387;p<0.001), whereas IP2 was positively associated with depression (β:0.14; p<0.001) and sleep problems (β:0.033; p<0.001). The indirect effect of IP2 on cognition, mediated by depression and sleep problems, was -0.043 and statistically significant. Model 2 indicates partial mediation by depression and sleep problems between indoor air pollution and cognitive health, resulting in explaining 45.5% of the variability. Finally, the third model with indoor air pollution considering poor cooking practices along with indoor smoke product use and secondhand smoke (IP3), was negatively associated with cognition (β:-0.381; p<0.001) and positively associated with depression (β:0.186; p<0.001) and sleep problem (β:0.038; p<0.001). The indirect effect of IP3 on cognition, mediated through depression and sleep problems, was -0.056, but the variability explained by the model remained the same at 45.5%. The models were also adjusted for socio-economic and demographic covariates selected within the framework of the study (Table 2).

**Table 2:** Multiple mediation analysis results of sleep problems and depression between indoor air pollution and cognitive health, LASI, 2017-2018

	**Depression(M1)**		**Sleep problems(M2)**		**Cognition(Y)**	
	*β*	*SE*	*β*	*SE*	*β*	*SE*
**Model 1:** Poor cooking practices and mental health						
**Indoor Air Pollution (X)**	0.158***	0.008	0.011**	0.006	-0.394***	0.026
**Depression(M1)**			0.153***	0.003	-0.237***	0.012
**Sleep problems (M2)**					-0.158***	0.017
**Age**	0.005***	0.001	0.009***	0	-0.114***	0.002
**MPCE quintile**	-0.024***	0.005	0.029***	0.003	0.194***	0.015
**Years of Schooling**	-0.030***	0.002	-0.010***	0.001	0.668***	0.005
**Sex**						
Male®						
Female	0.012	0.014	0.135***	0.01	-1.870***	0.045
**Residence**						
Rural®						
Urban	-0.053***	0.015	-0.055**	0.01	1.296***	0.045
**Religion**						
Other religious groups®						
Hindus	0.176***	0.015	0.084***	0.01	0.095**	0.046
**Caste**						
Other caste group ®						
SC/ST	-0.108	0.014	-0.097***	0.01	-0.847***	0.044
**Marital Status**						
Not married ®						
Currently married	-0.273***	0.017	-0.044***	0.012	0.425***	0.054
**R-square**	0.034		0.073		0.456	
**F-test**	250.962	*p*<0.001	505.794	*p*<0.001	4902.045	*p*<0.001
**Indirect Effect Pathways**						
**Indoor Pollution > Depression > Cognition**						-0.038***
**Indoor Pollution > sleep problem > Cognition**						-0.002***
**Indoor Pollution > Depression > sleep problems > Cognition**						-0.036***
**Model 2:** Poor cooking practices, indoor smoke product usage and mental health						
**Indoor Air Pollution (X)**	0.140***	0.01	0.033***	0.007	-0.387***	0.032
**Depression(M1)**			0.153***	0.003	-0.243***	0.012
**Sleep problems(M2)**					-0.156***	0.017
**Age**	0.005***	0.001	0.010***	0	-0.114***	0.002
**MPCE quintile**	-0.032***	0.005	0.030***	0.003	0.210***	0.015
**Years of Schooling**	-0.032***	0.002	-0.010***	0.001	0.673***	0.005
**Sex**						
Male®						
Female	0.006	0.015	0.136***	0.01	-1.857***	0.045
**Residence**						
Rural®						
Urban	-0.066***	0.015	-0.052***	0.01	1.321***	0.045
**Religion**						
Other religious groups®						
Hindus	0.169***	0.015	0.082***	0.01	0.116**	0.046
**Caste**						
Other caste group ®						
SC/ST	-0.096***	0.014	-0.098***	0.01	-0.875***	0.044
**Marital Status**						
Not married ®						
Currently married	-0.278***	0.017	-0.044***	0.012	0.436***	0.054
**R-square**	0.031		0.073		0.455	
**F-test**	231.882	*p*<0.001	507.677	*p*<0.001	4888.71	*p*<0.001
**Indirect Effect Pathways**						
**Indoor Pollution > Depression > Cognition**						-0.034***
**Indoor Pollution > Sleep problem > Cognition**						-0.005***
**Indoor Pollution > Depression > Sleep problems > Cognition**						-0.003***
**Model 3:** Poor Cooking practices, indoor smoke product usage, second-hand smoke and mental health						
**Indoor Air Pollution (X)**	0.186***	0.012	0.038***	0.008	-0.381***	0.037
**Depression(M1)**			0.153***	0.0028	-0.243***	0.012
**Sleep problems(M2)**					-0.157***	0.017
**Age**	0.005***	0.001	0.010***	0	-0.114***	0.002
**MPCE quintile**	-0.032***	0.005	0.030***	0.003	0.216***	0.015
**Years of Schooling**	-0.032***	0.002	-0.010***	0.001	0.673***	0.005
**Sex**						
Male®						
Female	0.01	0.015	0.136***	0.01	-1.858***	0.045
**Residence**						
Rural®						
Urban	-0.072***	0.015	-0.054***	0.01	1.351***	0.045
**Religion**						
Other religious groups®						
Hindus	0.171***	0.015	0.083***	0.01	0.105**	0.046
**Caste**						
Other caste group ®						
SC/ST	-0.099***	0.014	-0.098***	0.01	-0.878***	0.044
**Marital Status**						
Not married ®						
Currently married	-0.281***	0.017	-0.045***	0.012	0.442***	0.054
**R-square**	0.032		0.073		0.455	
**F-test**	237.632	*p*<0.001	507.621	*p*<0.001	4881.056	*p*<0.001
**Indirect Effect Pathways**						
**Indoor Pollution > Depression > Cognition**						-0.045***
**Indoor Pollution > Sleep problem > Cognition**						-0.006***
**Indoor Pollution > Depression > Sleep problem > Cognition**						-0.005***

®: Reference category; *** *p*<0.001, ** *p*<0.01, * *p*<0.05

**Fig. 4:**
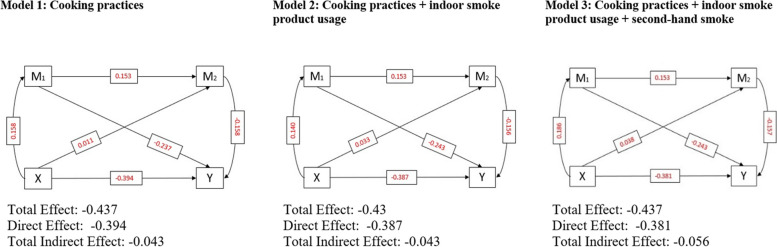
Pathways of the multiple-mediation models showing direct, indirect, and total effects

As observed from Fig. 4, with the inclusion of indoor air pollution index indicators, there was a slight variability in the mediation effect. In Model 1, where the indoor air pollution index construct included only poor cooking practices, the total effect on cognition was -0.437; i.e., cognition was reduced by 43.7%. The indirect effect of the mediation of depression and sleep problems brought about 9.8% variability in the total effect on cognition. Further, in Model 2, indoor air pollution was constructed by indoor smoke product usage along with poor cooking practices, we observed a total effect of -0.430, which was not too different from Model 1, but there was a slight increase in the mediation effect, which brought 10% variability in the total effect. Finally, Model 3, comprised of poor cooking practices, indoor smoke product usage, and secondhand smoke in the index, was similar to the former models in terms of the total effect, which in this case was -0.437. However, the indirect effect due to mediation by depression and sleep problems was higher in Model 3; 12.8% of the total effect on cognition was explained by the indirect effect (Fig. 4). Thus, we can conclude that Model 3 in (Fig. 4) showed a better picture of the mediation effect observed from the indirect effect of indoor air pollution on cognition through depression and sleep problems (-0.056). The variation in cognition is sensitive to changes in the construct of indoor air pollution.

## Discussion

The number of older adults is rising worldwide due to factors such as declining fertility, improved longevity, and medical gains [42]. While considered a young country demographically, the global social transition and population aging also have critical implications for India. Our study attempted to understand the spatial variation of different sources of indoor air pollution and their association with cognitive health. Further, we explored the pathway between indoor air pollution and cognition by testing mediating effects of depression and sleep problems among older adults in India. Multiple mediation analysis applied in the current study is an emerging statistical method in theory and practice. It is used in various research settings to understand causal relationships [43]. The major findings are discussed below:

First, indoor air pollution was higher in India's Northern and Eastern regions, and low cognitive scores were also observed in these regions. Traditional cooking practices such as solid biomass fuels like firewood, crop residue, cow dung, coal, etc., are more prevalent in the rural parts of northern and eastern India. Another major source of air pollution in these areas is agricultural biomass burning in the northern Indo-Gangetic plains and forest fires in the northeastern states, where a piece of land is cleared and burned for human use [44]. Thus, considering different sources of indoor and outdoor air pollution, India is the third-largest emitter of carbon emissions after China and the United States of America [45]. The use of incense sticks, also called agarbatti/dhoop, has been a part of Indian culture for centuries and is rooted in various traditions and rituals. The release of particulate matter into the air, including fine particles and smoke, impacts respiratory health, especially for individuals with pre-existing conditions such as asthma or allergies [46].

Based on the NSS 2011-2012, the monthly usage of incense sticks (agarbatti) and room freshener in urban and rural India stands at 742 and 737 per 1000 households, respectively [47]. Moreover, on average, Indian households burn mosquito coils for approximately 6-8 hours daily. The mosquito coils/fast cards contain insecticides and other toxic compounds, making them particularly hazardous, especially in poorly ventilated rooms [48, 49]. Per the Global Adult Tobacco Survey (GATS) (2016–2017), 29% of the Indian population was exposed to secondhand smoke; females are exposed at home, and males are more exposed in public and workplaces [50].

Second, our study generated three models to understand indoor air pollution exposure; we used different types of poor cooking practices, indoor smoke product usage, and second-hand smoke to characterize the country-specific context of indoor air pollution in India. The association between indoor pollution and mental health is evident. Most importantly, the study reveals that the direct effect of the multiple mediation model was amplified by the indirect effect making the total effect more significant, thus showing more robustness than a simple regression model. In addition, when combined, the three components of indoor pollution have the highest explanatory power, indicating that all three components of indoor pollution exposure play an important role in cognitive decline.

Third, we observe the indirect or mediation effect by serial mediation of sleep problems followed by depression. Such multiple mediation models have been studied previously but not in the context of indoor air pollution and cognition, particularly in the Indian context [51]. In the current study, the multiple mediation model first observes the direct exposure of indoor air pollution on cognition, which tends toward zero, suggesting a mediation effect [52]. Indoor pollution may disrupt sensitive brain structures and processes and have long-term negative impacts like depression and disrupted sleep patterns, particularly among the elderly [31, 53–56]. Therefore, in our study, we quantified the effect of mediators on the exposure variable (cognition).

In the growing scholarship on mechanisms and pathways through which air pollution is associated with cognitive health, we offer multiple points of consideration. The mediation analysis suggests that across all conceptualizations of indoor air pollution, depression, and sleep problems accounted for some but not all of the relationship between indoor air pollution and cognition. Therefore, while indoor air pollution was associated with cognitive decline, this relationship was mediated by a person experiencing depression and sleep problems, with sleep problems showing a stronger mediation effect. Our multiple mediation analysis demonstrates how the hypothesized mediators, depression and sleep problems, explain the effect of indoor air pollution on cognitive health. The construct of indoor air pollution, including all three dimensions, shows that mediating effects of sleep problems and depression further increase negative impacts of indoor air pollution on cognition. Since depression and sleep disorders can lead to cognitive decline among older adults, future investigations in communities exposed to higher pollution rates should examine the potential protective factors in safeguarding cognitive health among this population.

Our findings have particular policy implications for India. An analysis of the National Mental Health Survey in India points to higher prevalence of depressive disorders among older adults [57], and sleep disorders, including sleep problems, are common among this population [58]. India shoulders some of the highest global burden of disease attributable to air pollution; indoor air pollution remains an important factor therein [59] and therefore presents a key challenge for India’s commitment to sustainable development. Our results provide a regional and contextual understanding of indoor air pollution outcomes, in that unsafe poor cooking practices, indoor smoke from incense sticks and mosquito coils, and secondhand smoking are associated with lower sleep quality and depression among India's older adult population. North and East India can benefit the most by addressing indoor pollution. Second, our results confirm the negative association of indoor air pollution with cognitive health in all models. This is consistent with other studies examining the association of air pollution with cognitive health [1, 15, 19]; e.g., Exposure to indoor PM_2.5_ might contribute to 476,000 cases of major depressive disorder in the U.S. [60] and a decline in cognitive function among older men owing to exposure to black carbon [61]. The home and built environment can significantly improve resident mental health; considering the converging challenges of mental health, indoor air pollution, and other ecological factors, therefore, is a policy imperative to achieve Sustainable Development Goal 3 (SDG-3), by enhancing good health and well-being of all which is intricately linked with climate change (SDG 13).

Some limitations should be considered to contextualize our findings. While LASI is designed to be a longitudinal survey, data for this study are from the first wave. Therefore, these data are cross-sectional, and the findings do not lend to causal inferences. However, our analysis provides a useful baseline on a critical issue that can be extended with the availability of subsequent waves and longitudinal studies in the future.

## Conclusion

The global burden of disease from indoor air pollution and the mounting, relatively unaddressed mental health crisis in India necessitates the transition to clean fuel and expansion of mental health services*.* Multitudes in India face challenges of poor indoor air quality, especially in North, East, and North-Eastern parts of India, from varying sources, including unhealthy cooking fuels, burning of different household products, and tobacco smoking. Sleep quality is critical for improving mental health and reducing depression among older adults; this is especially relevant when evaluating fuel and burning product choices within the home and the built environment. Our study provides the basis for further research into the processes of indoor air pollution, the specific types of fuels used for heating and cooking, insufficient ventilation in the home, and other smoke exposure. In examining the association between air pollution and cognition, our study lays the foundation for future investigations into this nexus to inform and formulate policies to reduce exposure to air pollutants and improve screening and access to mental health services as a public health priority.

### Supplementary Information

**Additional file 1: Table S1.** Descriptive statistics of the study variables (*N*= 72,250).

## Data Availability

The data is freely available upon request from https://www.iipsindia.ac.in/content/LASI-data.
