# Supplementary material for: Indoor air pollution and cognitive function among older adults in India: a multiple mediation approach through depression and sleep disorders
Source: BMC Geriatr. 2024 Jan 22;24:81. doi: 10.1186/s12877-024-04662-6 (PMC10802029; doi:10.1186/s12877-024-04662-6)
Supplement: Supplementary file 1 — Additional file 1: Table S1. Descriptive statistics of the study variables (N= 72,250). [file 12877_2024_4662_MOESM1_ESM.docx]

**Title: Indoor air pollution and cognitive function among older adults in India: A multiple mediation approach through depression and sleep disorders**

**Table S1:** Descriptive statistics of the study variables (N= 72,250)

| Covariates |  | N (%) | Mean (S.D) | Range |
| --- | --- | --- | --- | --- |
| Gender | Male | 30342(42) |  |  |
|  | Female | 41908(58) |  |  |
| Age |  |  | 58.57(11.82) | 18-116 |
| Residence | Rural | 49274(68.2) |  |  |
|  | Urban | 22976(31.8) |  |  |
| Education |  |  | 4.06(4.94) | 0-26 |
| Religion | Hindu | 59188(81.92) |  |  |
|  | Others | 13062(18.08) |  |  |
| Marital status | Married | 54622(75.6) |  |  |
|  | Not married | 17628(24.4) |  |  |
| Caste | SC/ST | 13833(19.15) |  |  |
|  | Others | 52251(72.32) |  |  |
| MPCE | Poorest | 14956(20.7) |  |  |
|  | Poorer | 15328(21.22) |  |  |
|  | Middle | 14790(20.47) |  |  |
|  | Richer | 14151(19.59) |  |  |
|  | Richest | 13025(18.03) |  |  |
|  |  |  |  |  |
| **Independent variable (X)** |  |  |  |  |
| Indoor Pollution [Cooking practices] (IP1) |  |  | 3.65(3.27) | 0-10 |
| Indoor Pollution [Cooking practices + indoor smoke product usage] (IP2) |  |  | 2.12(1.71) | 0-10 |
| Indoor Pollution [Cooking practices + indoor smoke product usage + second-hand smoke] (IP3) |  |  | 2.06(1.69) | 0-10 |
| **Mediators** |  |  |  |  |
| Depression (M1) |  |  | 2.94(1.7) | 0-10 |
| Insomnia (M2) |  |  | 0.79(1.24) | 0-4 |
| **Dependent variable (Y)** |  |  |  |  |
| Cognition |  |  | 25.38(6.79) | 3-43 |
